# Development of a new occupational balance-questionnaire: incorporating the perspectives of patients and healthy people in the design of a self-reported occupational balance outcome instrument

**DOI:** 10.1186/1477-7525-12-45

**Published:** 2014-04-05

**Authors:** Mona Dür, Günter Steiner, Veronika Fialka-Moser, Alexandra Kautzky-Willer, Clemens Dejaco, Birgit Prodinger, Michaela Alexandra Stoffer, Alexa Binder, Josef Smolen, Tanja Alexandra Stamm

**Affiliations:** 1 Department of Internal Medicine III, Division of Rheumatology, Medical University of Vienna, Währinger Gürtel 18-20, Vienna 1090, Austria; 2 Department of Physical Medicine and Rehabilitation, Medical University of Vienna, Währinger Gürtel 18-20, Vienna 1090, Austria; 3 Department of Internal Medicine III, Division of Diabetology, Medical University of Vienna, Währinger Gürtel 18-20, Vienna 1090, Austria; 4 Department of Internal Medicine III, Division of Gastroenterology and Hepatology, Medical University of Vienna, Währinger Gürtel 18-20, Vienna 1090, Austria; 5 Swiss Paraplegic Group, ICF Unit, Guido A. Zäch Straße 4, Nottwil 6207, Switzerland; 6 Department of Occupational Therapy, Hospital Göttlicher Heiland, Dornbacherstraße 20-28, Vienna 1170, Austria; 7 Hospital Hietzing and Neurological Centre Rosenhügel, Wolkersbergenstraße 1, Vienna 1130, Austria; 8 Department of Health, University of Applied Sciences, FH Campus Wien, Favoritenstraße 226, Vienna 1100, Austria

**Keywords:** Qualitative research, Rasch analysis, Occupational science, Patient perspective

## Abstract

**Background:**

Self-reported outcome instruments in health research have become increasingly important over the last decades. Occupational therapy interventions often focus on occupational balance. However, instruments to measure occupational balance are scarce. The aim of the study was therefore to develop a generic self-reported outcome instrument to assess occupational balance based on the experiences of patients and healthy people including an examination of its psychometric properties.

**Methods:**

We conducted a qualitative analysis of the life stories of 90 people with and without chronic autoimmune diseases to identify components of occupational balance. Based on these components, the *Occupational Balance-Questionnaire* (*OB-Quest*) was developed. Construct validity and internal consistency of the *OB-Quest* were examined in quantitative data. We used Rasch analyses to determine overall fit of the items to the Rasch model, person separation index and potential differential item functioning. Dimensionality testing was conducted by the use of t-tests and Cronbach’s alpha.

**Results:**

The following components emerged from the qualitative analyses: *challenging and relaxing activities*, *activities with acknowledgement by the individual and by the sociocultural context*, *impact of health condition on activities*, *involvement in stressful activities and fewer stressing activities*, *rest and sleep*, *variety of activities*, *adaptation of activities according to changed living conditions* and *activities intended to care for oneself and for others*. Based on these, the seven items of the questionnaire (OB-Quest) were developed. 251 people (132 with rheumatoid arthritis, 43 with systematic lupus erythematous and 76 healthy) filled in the *OB-Quest*. Dimensionality testing indicated multidimensionality of the questionnaire (t = 0.58, and 1.66 after item reduction, non-significant). The item on the component *rest and sleep* showed differential item functioning (health condition and age). Person separation index was 0.51. Cronbach’s alpha changed from 0.38 to 0.57 after deleting two items.

**Conclusions:**

This questionnaire includes new items addressing components of occupational balance meaningful to patients and healthy people which have not been measured so far. The reduction of two items of the *OB-Quest* showed improved internal consistency. The multidimensionality of the questionnaire indicates the need for a summary of several components into subscales.

## Background

The use of self-reported outcome instruments in health care research has become increasingly important over the last decades, because the perspective of patients is an essential part regarding the effectiveness or non-effectiveness of a treatment [1-3]. Currently, it is recommended not only to measure outcomes from the perspectives of the patients, but also to involve them into the development of instruments. Patients should be asked for feedback on the wording to detect problems of understanding. Additionally, for already existing instruments, qualitative research is suitable in facilitating that the items are relevant to the target population [4-6].

Occupational balance largely guides the clinical practice of occupational therapists [7,8]. "Occupations" refer to goal-directed, meaning- and purposeful everyday activities that people do as individuals and in their social contexts [7]. Occupational therapists focus on occupations as a means, but also as an outcome of therapy. Occupational balance is one important construct that links – in the view of occupational therapists – "occupation" and health [9,10].

Occupational balance is defined diversely. One definition which is grounded in the beginning of occupational therapy refers to a balance between different occupational areas, such as work, play, rest and sleep [7]. However, the definitions are mainly derived from the perspectives of occupational therapists rather than of the perspectives of patients and healthy people (without a diagnosed health condition) [11]. Occupational balance may thus be an "academically defined concept" which lacks a link to the experiences of "real" people.

Up to now, only one questionnaire exists (Wilcock’s two-page "questionnaire on involvement in physical, mental, social and rest occupations") which was developed and used to assess occupational balance [12]. However, this questionnaire has not been developed based on qualitative data, and was not used in further research. One essential aspect of the validity of an instrument is the content validity, referring to its ability to measure those underlying components of the construct which it intends to measure. This requires a conceptual definition of the construct to be measured and a specification of its components [13]. To assess occupational balance in patients of different health conditions, a generic self-reported outcome instrument, based on qualitative data on the perspectives and experiences of patients and healthy people, is required. Additionally, reliable and valid (occupational balance) instruments are prerequisites for the evaluation of outcomes in occupational therapy practice.

Therefore, the aim of the study was to develop a generic self-reported outcome instrument to assess occupational balance based on the experiences of patients and healthy people including an examination of its psychometric properties.

## Methods

### Design

**Figure 1:**
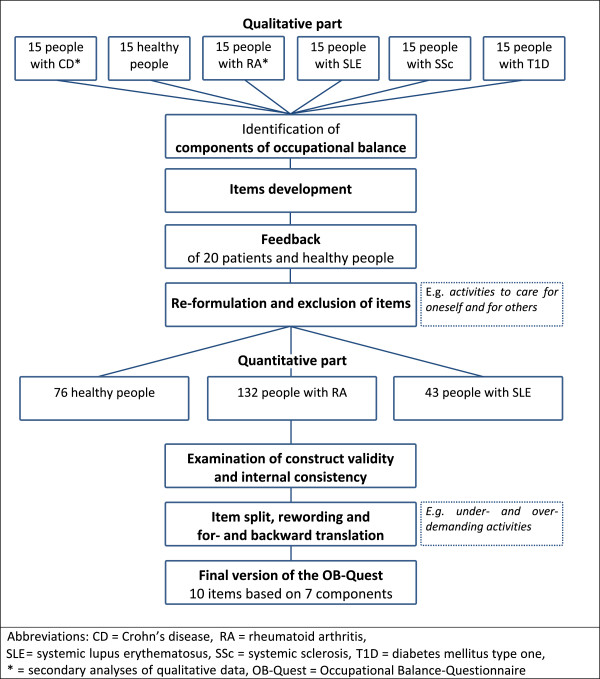
Flow chart: Process of the development of the Occupational Balance-Questionnaire.

We conducted a mixed-methods study that started off with qualitative analyses of the life stories of people with and without chronic autoimmune diseases to identify components of occupational balance. Based on these components, we developed the *Occupational Balance-Questionnaire* (*OB-Quest*). A German version was designed first and then forward and back translated into English according to standard translation procedures [14]. Construct validity and internal consistency of the *OB-Quest* were examined in quantitative data using Rasch analyses and Cronbach’s alpha. This study was part of a larger study, the Gender, Occupational Balance and Immunology (GOBI) study [15]. A flow chart is depicted in Figure 1.

### Participants

Patients of two outpatient clinics of the Medical University of Vienna, Austria, diagnosed with Crohn’s disease (CD) [16], rheumatoid arthritis (RA) [17], systemic lupus erythematosus (SLE) [18], systemic sclerosis (SSc) [19] or diabetes mellitus type one (T1D) [20] were asked to participate. Only the qualitative data from patients with CD and RA were used from previous studies which employed the same methodological approach [11,15,21]. Additionally, healthy people were asked to participate via personal invitation by patients, such as "friends" of a similar age, and announcements in public places such as supermarkets, universities and the general hospital. Sex, age, employment status and health condition, if applicable were recorded. Furthermore, information about disease duration was obtained from patient files where appropriate.

### A qualitative analysis of life stories with relation to occupational balance

A biographical narrative approach was used to explore the experience of occupational balance. The participants’ own perspectives on their lives, expressed in their life stories, were investigated in relation to current and biographic experiences [22]. The life stories, collected in two interview sessions, were transcribed verbatim and analysed with the biographic narrative interpretative method [22]. With this method, we compered the "lived life" (the biographical data) to the "told story" (the present perspective of the interviewee on her or his life). Hypotheses were developed by a research panel which were then verified or falsified depending on the course of each life story. Through this process, so-called typologies, which were common themes found in more than one life story, were identified. Typologies related to occupational balance were then extracted, defined as components of occupational balance and used as basis for the development of the questionnaire items.

### Item development

Each component resulted in one item. Items were generated by the first author in collaboration with patients and healthy people [4]. After this initial process a final version of the items was formulated based on the feedback of additional patients and healthy people and on the discussion of the research panel (TAS, BP, AB and MD). Patients from the rheumatology outpatient clinic and internal medicine ward, as well as healthy people ("friends" of a similar age and visitors) were invited to give feedback. Each item included a numerical rating scale consisting of three response categories. "1" indicated a positive score, such as "having a good variety of activities", and "3" indicated a negative score such as "having little or no variety of activities". In addition, a German version was designed first and then the English version was developed by the use of a standard methodology with forward and back translation and proof reading by a total of four English or German native speakers (PG, AJ, LL and VN-D) [14].

### Examination of construct validity and internal consistency

Construct validity and internal consistency of the *OB-Quest* were explored. Therefore, patients with RA or SLE and healthy people completed the *OB-Quest*, as well as a questionnaire on demographic data. The Statistical Package for the Social Sciences (SPSS) [23] was used for descriptive and RUMM 2030 [24] for Rasch analyses. Concerning construct validity we examined overall fit of the items to the Rasch model as suggested by Tennant et al. [25]. Therefore, the mean item log residual test of fit as well as the item-trait interaction chi-square statistics were assessed [25]. Non-significant residuals between -2.5 and +2.5 were interpreted as item fit, non-significant item-trait interaction chi-square values as overall fit [25-27]. Additionally, we calculated potential differential item functioning (DIF) of sex, age (above and below the median) and health condition (RA, SLE and healthy) concerning construct validity. Furthermore, we used an approach for unidimensionality testing proposed by Smith [28], namely the combination of principal component analysis (PCA) followed by a series of t-tests to assess if subsets of items result in different estimates of person parameters. As prerequisites for the t-tests, "easy" and "hard" subsets of items were selected based on PCA. The sets of items whose residual factors loaded most strongly (positively or negatively) on the first principal component factor were used because these are most likely to violate the assumption of unidimensionality. Easy items were defined as having fit residuals that loaded negatively on the first component [29]. We used Bonferroni adjustment for multiple testing regarding the level of significance of the results of the Rasch analyses. Internal consistency is an estimate of an instruments’ reliability and was assessed with Cronbach’s alpha (α) and Rasch reliability statistics (person separation index, PSI). PSI refers to the reproducibility of relative measure location. A high PSI value (≥ 0.7) is preferred, since this indicates a high probability that people with enhanced performance will achieve high measures (sensitivity) and vice versa [30,31]. We interpreted Cronbach’s α of ≥ 0.9 as excellent, ≤ 0.89 and ≥ 0.8 as good, ≤ 0.79 and ≥ 0.7 as acceptable and ≤ 0.69 and ≥ 0.6 as questionable [23].

### Ethical considerations

Participants were informed about study procedures, and confirmed their voluntary participation with written and oral informed consents. Furthermore, we guaranteed confidentiality and changed the names in the given quotes. Approval of the Ethics Committee of the Medical University of Vienna, Austria, was obtained.

## Results

### Participants

Ninety people participated in the qualitative part of this study (15 people each with CD, SLE, SSc, RA or T1D and 15 healthy people). The data of 251 people (132 people with RA, 43 with SLE and 76 healthy people) were collected in the quantitative part of this study. Demographic characteristics of the participants are shown in Table 1.

**Table 1:** Demographic characteristics of the participants

	**Qualitative study**						**Validation study**		
**Characteristics**	**Health conditions**						**Health conditions**		
	**Crohn’s disease**	**Diabetes mellitus type 1**	**Healthy**	**Rheumatoid arthritis**	**Systemic lupus erythematosus**	**Systemic sclerosis**	**Healthy**	**Rheumatoid arthritis**	**Systemic lupus erythematosus**
*n* participants	15	15	15	15	15	15	76	132	43
*n* (%)									
Female	8 (53)	8 (53)	10 (67)	11 (73)	9 (53)	11 (73)	48 (63)	116 (88)	41 (95)
Employed^+^	8 (53)	8 (53)	11 (73)	0	10 (67)	4 (27)	65 (86)	42 (32)	21 (49)
β-Blocker/ACE inhibitors/statins	1(7)	6 (40)	--	3 (20)	9 (60)	9 (60)	--	43 (35)	17 (46)
DMARD	7 (47)	--	--	15 (100)	9 (60)	2 (13)	--	70 (54)	32 (76)
Biologic	5 (33)	--	--	8 (47)	0	0	--	54 (42)	0
ASA	9 (60)	--	--	--	--	--	--	--	--
Cortisone	--	--	--	5 (33)	10 (67)	4 (27)	--	47 (36)	26 (63)
Low disease activity/remission	11 (73)^a^	3 (27)^b^	--	7 (47)^c^	1 (7)^d^	10(67)^e^	--	96 (72)^c^	36 (83)^e^
Median (IQR)									
Age	46	53	36	54	46	67	38	59	46
	(34-60)	(36-63)	(32-62)	(43-58)	(32-60)	(50-74)	(27-49)	(48-65)	(36-53)
Disease duration	15	20	--	11	10	10	--	10	10
	(8-30)	(11-40)		(9-20)	(8-19)	(4-16)		(4-16)	(8-17)

*Abbreviations*: *n* number, *(%)* percentage, *+* employed/self-employed, *β-Blocker/ACE inhibitors/statins* took Beta-blocker, ACE-inhibitors and/or statins, *DMARD* disease-modifying anti-rheumatic drugs, *Biologic* biologic DMARDs, ^a^Harvey Bradshaw Index (HBI) [32], ^b^excellent glycosylated haemoglobin below 53 millimoles per litre (mmol/l, 7.0%, Hba1c) [33], ^c^Clinical Disease Activity Index (CDAI) [34], ^d^Systemic Lupus Erythematosus Disease Activity Index (SLEDAI) [35,36], ^e^Rodnan skin score = total remission < 10 (RODNAN) [37], *IQR* interquartile range, -- not applicable.

### Components of occupational balance

Eight components of occupational balance were identified in the analysis of the qualitative data, as described in Table 2 in the left column. In the following we give one example of how the components relate to the transcribed data. Sabine was diagnosed with systemic sclerosis at the age of 59 years. Sabine maintained occupational balance through a balance of *challenging and relaxing activities*:

*This year I was tandem parachuting four times, out of an airplane of 4000 meters height* (first interview, lines 170-171). *I love such challenges. I need something like this* [parachuting, long distance motor cycling] *in addition to my daily* routine (first interview, lines 223-224).

*Yesterday, we have played tennis. I must say that afterwards I need some relaxation* (second interview, lines 330-331).

**Table 2:** Item development of the occupational balance-questionnaire

**Components of occupational balance**	**Items for the OB-quest**	**Suggested items for the revised OB-quest**
1. Challenging and relaxing activities	1. In your daily life, are there occupations and activities that you find challenging?	1. Do you generally find your activities in your everyday life under-demanding?
		2. Do you generally find your activities of daily living over-demanding?
2. Activities with acknowledgement by the individual and by the sociocultural context	2. Are there occupations and activities for which you receive acknowledgement?	3. Do you generally receive enough appreciation for activities in your everyday life?
3. Involvement in stressful activities and fewer stressing activities	3. Please think about all your occupations and activities – are there periods in which you feel overstressed?	4. How often do you feel overstressed in your everyday life?
4. Impact of own health condition on activities	4. In your day to day activities, do you feel affected by your health?	5. How much are your activities in your everyday life affected by your health?
5. Satisfaction with the amount of rest and sleep	5. How satisfied are you with the amount of rest and/or sleep that you get?	6. Do you get enough rest?
		7. Do you get enough sleep?
6. Engagement in a variety of activities	6. Do you have a good variety of different occupations and activities that you do? For example, do you do a mixture of physical activities and more sedentary ones (where you are sitting down or staying still)? Or a mixture of creative activities and activities that are more	8. Do you have sufficient variety of different activities that you do? For example, do you do a mixture of physical activities and more sedentary ones (where you are sitting down or staying still)? Or a mixture of creative activities and activities that are more routine for you?
7. Adaptation of activities according to changed living conditions, such as a chronic autoimmune disease or changes in work or family circle	7. How do you rate your ability to adapt your occupations and activities to changing living conditions (e.g. changes in your health, or work)?	9. How well can you adapt your activities in your everyday life to changed living conditions, such as a changed state of health?
		10. How well can you adapt your activities in your everyday life to changed living conditions, such as a change of your professional life or employment status?
8. Activities intended to care for oneself and for others		

### Occupational Balance-Questionnaire (OB-Quest)

The eight components of occupational balance identified in the qualitative analysis were used for the development of the questionnaire items, as shown in Table 2.

The first draft of the *OB-Quest* was piloted in 20 additional patients and healthy people. Based on their feedback some questions were reworded. The item *activities intended to care for oneself and for others* was deleted because patients complained that this question would not be relevant for those who did not care for others. The same applied to the example *changed family circle* in the item on the component *adaptation of activities according to changed living conditions*. Finally, seven items were formulated as presented in Table 2, second column.

### Construct validity and internal consistency

The results of the overall fit, item and fit statistics referring to construct validity are shown in Table 3.

**Table 3:** **Overall fit, item and fit statistics of the Rasch analyses for the *occupational balance-questionnaire***

**Overall fit statistics**	**Chi-square *p*** **≤ 0.01 35.15**		**Chi-square**^**red **^ ***p*** **≤ 0.01 11.20**	**ẍ item fit residual (± SD) 0.54 (± 1.71)**	**ẍ item fit residual**^**red **^**(± SD) 1.69, (± 0.89)**
**Item and fit statistics**	**Item statistics**^**a**^		**Fit statistics**^**a**^		
**Components of items**	**Location (Location**^**red**^**)**	**SE (SE**^**red**^**)**	**Residual (Residual**^**red**^**)**	**Chi-Square (Chi-square**^**red**^**)**	**F-statistic (F-statistic**^**red**^**)**
Challenging and relaxing activities	0.216	0.084	**3.395**	12.665*	12.784*
	--	--	--	--	--
Activities with acknowledgement by the individual and by the sociocultural context	0.025	0.077	0.340	2.248	2.719
	(0.101)	(0.080)	(2.801**)	(0.267)	(0.048)
Involvement in stressful activities and fewer stressing activities	-0.397	0.078	0.082	2.527	3.211
	(-0.302)	(0.082)	(1.584)	(2.324)	(2.183)
Impact of health condition on activities	-0.156	0.073	0.213	1.241	1.750
	(-0.055)	(0.075)	(0.519)	(6.352)	(8.439)
Rest and sleep	-0.230	0.073	1.522	0.181	0.103
	(-0.141)	(0.076)	(2.276)	(0.447)	(0.126)
Variety of activities	0.302	0.075	0.502	1.023	0.489
	(0.397)	(0.077)	(1.264)	(1.815)	(1.383)
Adaptation of activities according to changed living conditions	0.240	0.073	-2.284	15.264*	27.360*
	--	--	--	--	--

^a^rounded to three decimals.

Location = Expressed in linear log-odds units (logits). Mean item location is zero with positive values representing more limitations of occupational balance.

^red^without items on the components *challenging and relaxing activities* and *ability to adapt activities*, after item deletion.

SE = Standard error.

Residuals = summarize the deviation of observed from expected responses. Deviation from the recommended range of -2.5 to +2.5 indicating misfit are bold.

Chi-square = Chi-square values summarize the deviation of observe from expected responses across the three class intervals of people. Higher values indicate larger deviations.

F-statistics = F-statistics from one-way Analysis of variance of deviations from model expectation across the three class intervals of people.

*Bonferroni corrected statistically significant deviations across class intervals, indicating misfit (bold, Bonferroni adjusted probability level = .001429).

**Bonferroni corrected statistically significant deviations across class intervals, indicating misfit (bold, Bonferroni adjusted probability level = .002000).

The following sets of items whose residual factors loaded most strongly were used for t-tests: *variety of activities*, *adaptation of activities according to changed living conditions*, *impact of own health condition on activities*, *involvement in stressful and fewer stressful activities* (negative loading), and *challenging and relaxing activities*, *activities with acknowledgement by the individual and by the sociocultural context* and *rest and sleep* (positive loading). The result of the t-test disproved the equivalence of test scores within the *OB-Quest* (t = 0.58, non-significant) and thus confounded our hypothesis on unidimensionality of the questionnaire. The item on the component *rest and sleep* showed DIF regarding the categories health condition and age. Furthermore, PSI was 0.51 and Cronbach’s α 0.38, revealing low internal consistency. Therefore, we decided to remove the items on the components *challenging and relaxing activities* and *adaptation of activities according to changed living conditions*.

After deleting these items (components *challenging and relaxing activities* and *adaption of activities to changed living conditions*) the t-test and Cronbach’s α changed. The new results of the t-test (t = 1.66, non-significant) were based on the use of the sets of items on the components *involvement in stressful and fewer stressful activities* and *impact of health condition on activities* (positive loading), *activities with social acknowledgement*, *rest and sleep* and *variety of activities* (negative loading). Furthermore, Cronbach’s α changed to 0.57 indicating an improved internal consistency.

Because the item on the component *challenging and relaxing activities* confounded the unidimensionality of the *OB-Quest*, we decided to split this item into two concepts: too much and too little demand. Consequently, the two new items read now as follows: "1. Do you generally find your activities in your everyday life under-demanding? 2. Do you generally find your activities in your everyday life over-demanding?" Additionally, the item on the component *adaptation of activities to changed living conditions* misfitted the model. Accordingly this item was split into two concepts also, as shown in Table 2 right column. Furthermore due to DIF (as described above), the item on the component *rest and sleep* was divided into two items: one on *rest* and the other on *sleep*. Additionally, several changes were made based on the feedback of patients, e.g. particular words were reformulated (difficult to understand and/or score; Table 2). The final OB-Quest is proposed as ten items addressing seven components. Subsequently, the revised German version was forward and back translated; the final English and German versions of the *OB-Quest* are presented in Tables 4 and 5, respectively.

**Table 4:** **English version of the *Occupational Balance-Questionnaire *** **(OB-Quest)**

© Dür, Steiner, Fialka-Moser, Kautzky-Willer, Dejaco, Prodinger, Stoffer, Binder, Smolen & Stamm, 2014. Correspondence: tanja.stamm@meduniwien.ac.at			
**Occupational Balance-Questionnaire (OB-Quest)**			
"Occupations" or "activities" refer to all the things that you do, including very simple things, such as bathing or getting dressed. The definition of "activities" includes professional actions, free-time and relaxation activities (such as reading or sleeping), as well as childcare and the support of dependents. Please put an ‘x’ next to the *most applicable* answer to each question.			
1. Do you generally find your activities in your everyday life under-demanding?			
	I don’t find my activities to be under-demanding	I find some of my activities to be under-demanding	I find most of my activities to be under-demanding
	□	□	□
2. Do you generally find your activities in your everyday life over-demanding?			
	I don’t find my activities to be over-demanding	I find some of my activities to be over-demanding	I find most of my activities to be over-demanding
	□	□	□
3. Do you generally receive enough appreciation for activities in your everyday life?			
	I receive quite a lot of appreciation	I receive enough appreciation	I do not receive any appreciation
	□	□	□
4. How often do you feel overstressed in your everyday life?			
	Never	Sometimes	Often
	□	□	□
5. How much are your activities in your everyday life affected by your health?			
	Not at all	A little	Very much
	□	□	□
6. Do you get enough rest?			
	I get enough rest	I get little rest	I get very little rest
	□	□	□
7. Do you get enough sleep?			
	I get enough sleep	I get little sleep	I get very little sleep
	□	□	□
8. Do you have sufficient variety of different activities that you do? For example, do you do a mixture of physical activities and more sedentary ones (where you are sitting down or staying still)? Or a mixture of creative activities and activities that are more routine for you?			
	I have a sufficient variety	I have a little variety	I have no variety at all
	□	□	□
9. How well can you adapt your activities in your everyday life to changed living conditions, such as a changed state of health?			
	Very well	Badly	Not at all
	□	□	□
10. How well can you adapt your activities in your everyday life to changed living conditions, such as a change of your professional life or employment status?			
	Very well	Badly	Not at all
	□	□	□
**Thank you for completing the survey!**			

**Table 5:** **German version of the *Occupational Balance-Questionnaire *** **(OB-Quest)**

© Dür, Steiner, Fialka-Moser, Kautzky-Willer, Dejaco, Prodinger, Stoffer, Binder, Smolen & Stamm, 2014. Korrespondenz: tanja.stamm@meduniwien.ac.at			
**Fragebogen zu Ihrer Betätigungsbalance**			
"Betätigung" oder "Tätigkeit" bezieht sich auf alle Dinge, die Sie tun. Auch ganz einfache Dinge, wie z.B. sich selbst waschen oder anziehen. Aber natürlich auch auf Ihre berufliche Tätigkeit, Freizeit- und Erholungsaktivitäten (z.B. lesen oder schlafen), die Betreuung von Kindern, Enkelkindern, Angehörigen. Bitte kreuzen Sie *die am zutreffendste* Antwort zu jeder Frage an.			
1. Finden Sie Ihre Tätigkeiten in ihrem Alltag generell als unterfordernd?			
	Ich finde keine meiner Tätigkeiten als unterfordernd.	Ich finde einige meiner Tätigkeiten als unterfordernd.	Ich finde die meisten meiner Tätigkeiten als unterfordernd.
	□	□	□
2. Finden Sie Ihre Tätigkeiten in ihrem Alltag generell als überfordernd?			
	Ich finde keine meiner Tätigkeiten als überfordernd.	Ich finde einige meiner Tätigkeiten als überfordernd.	Ich finde die meisten meiner Tätigkeiten als überfordernd.
	□	□	□
3. Erhalten Sie ausreichend Anerkennung für Ihre Tätigkeiten in Ihrem Alltag?			
	Ich erhalte sehr viel Anerkennung	Ich erhalte ausreichend Anerkennung	Ich erhalte gar keine Anerkennung
	□	□	□
4. Wie oft erleben Sie ihren Alltag als zu stressüberladen?			
	Nie	Manchmal	Oft
	□	□	□
5. Wie beeinträchtigt Sie Ihr Gesundheitszustand in Ihren Tätigkeiten in Ihrem Alltag?			
	Gar nicht	Wenig	Sehr
	□	□	□
6. Haben Sie ausreichend Ruhe?			
	Ich habe ausreichend Ruhe	Ich habe wenig Ruhe	Ich habe sehr wenig Ruhe
	□	□	□
7. Haben Sie ausreichend Schlaf?			
	Ich habe ausreichend Schlaf	Ich habe wenig Schlaf	Ich habe sehr wenig Schlaf
	□	□	□
8. Haben Sie ausreichend Abwechslung von verschiedenen Tätigkeiten, die Sie durchführen? (Zum Beispiel: Haben Sie eine Mischung von aktiven Tätigkeiten und Tätigkeiten, bei denen Sie z.B. hauptsächlich sitzen, oder still stehen)? Oder eine Mischung aus kreativen Tätigkeiten und Tätigkeiten bei denen Sie mehr nach Vorgabe handeln?			
	Ich habe sehr viel Abwechslung	Ich habe wenig Abwechslung	Ich habe keine Abwechslung
	□	□	□
9. Wie gut können Sie Ihre Tätigkeiten im Alltag an veränderte Lebensumstände anpassen, wie beispielsweise eine Veränderung des Gesundheitszustandes?			
	Sehr gut	Schlecht	Überhaupt nicht
	□	□	□
10. Wie gut können Sie Ihre Tätigkeiten im Alltag an veränderte Lebensumstände anpassen, wie beispielsweise eine Veränderung ihrer Berufstätigkeit oder des Beschäftigungsstatus?			
	Sehr gut	Schlecht	Überhaupt nicht
	□	□	□
**Vielen Dank für das Ausfüllen des Fragebogens!**			

## Discussion

This article describes the development of a new self-reported outcome instrument to assess occupational balance. Item development was based on an exploration of occupational balance in qualitative data and the involvement of patients with chronic autoimmune diseases and healthy people. Furthermore we validated and revised the *OB-Quest* based on quantitative data of patients with RA or SLE and healthy people.

The *OB-Quest* includes items which have not been covered so far by instruments used to assess occupational balance. Challenging and relaxing activities and adaptation of activities have already been identified as components of occupational balance [21,38,39]. Additionally, stress has also been related to occupational balance earlier [40]. However, the four components *challenging and relaxing activities*, *involvement in stressful activities and fewer stressing activities, impact of health condition on activities*, and *adaptation of activities to changed living conditions* have not been included in occupational balance instruments.

Different instruments have been used to assess single components of occupational balance, as identified in the current study. For example, the Short-Form 36-items Health Survey (SF-36) [41] assesses, besides other aspects, the impact of a health condition on activities of daily living (questions on health limiting daily activities). Another example is the *Adolescent Stress Questionnaire* which includes items on having enough time for activities others than school tasks [42]. Frequently used instruments, such as the *Perceived Stress Questionnaire*[43] or the *Perceived Stress Scale*[44] do not capture potential relations between stress and activities. However, a "composition" of the seven components, identified in our study within one instrument, has not been developed.

The results of this study indicate that occupational balance might be a multidimensional construct. Additionally, the results of the t-tests (0.58 and 1.66, respectively), indicated that the test scores vary within the *OB-Quest* items. Furthermore, the authors of previous studies came to the conclusion that occupational balance might be a multidimensional construct [45,46]. Moreover, the two models of occupational balance which are based on empirical quantitative and qualitative data [10,47] also challenge a unidimensional conceptualisation of occupational balance. The multidimensionality indicates that a total score of the instrument is not justified and thus requests the summary of several of the components into subscales, as this is the case in the SF-36 [41]. Further research including components analysis is suggested to verify the various components of the OB-Quest.

The concept of "occupational imbalance" should be addressed in further research. Studies of occupational imbalance are scarce and findings are diverse [48]. For example, occupational imbalance, defined as interference among occupations, was negatively associated with subjective wellbeing [49] and life satisfaction [45]. However, this was not the case in another study on occupational imbalance [48]. Currently, it is unclear whether occupational balance and occupational imbalance represent opposite poles of one single dimension or two distinct concepts [45,48].

The component *activities intended to care for oneself and for others* is important for the maintenance of occupational balance in people who have someone they care about. When assessing occupational balance in people who do care for others, the following question could be used: "Could you take sufficient care of yourself while caring for another (such as a family member, loved one, etc.)?" The inclusion of optional items was found to be justified in specific settings or circumstances [6]. However, due to the feedback of patients and healthy people, we decided to exclude this item of the *OB-Quest*.

### Strengths and limitations

The development together with patients and healthy people, as well as the base on experiences of people with and without chronic autoimmune diseases strengthens the questionnaire’s construct validity [5,13]. Additionally, this process followed common recommendations for the development of self-reported outcome instruments [5,50] and resulted in the identification of new components of occupational balance. Cronbach’s α or the internal consistency of the questionnaire turned out to improve by the reduction of the items on the components *challenging and relaxing activities* and *adaptation of activities according to changed living conditions*. In the current study three of the four native speakers were without a health professional or patient perspective and conducted the forward and back translations of the different questionnaire versions. Another study could include patients of various diagnoses according to the International Classification of Diseases [19]. Moreover, a translation into different languages and an examination of the instrument’s psychometric properties in different countries all over the world, could allow an evaluation of its culture fairness. Additionally, a conceptual definition of occupational balance would be of great value and importance. We extracted components of occupational balance from qualitative data. However, we did not aim to provide a conceptual definition of occupational balance. The conceptualization of a concept requires a complex process of synthesizing the findings of several studies [51]. We suggest further studies on the conceptualization of occupational balance.

## Conclusions

This questionnaire includes new items addressing components of occupational balance meaningful to patients and healthy people which have not been measured so far. The *OB-Quest* showed improved internal consistency after the reduction of two items, based on data obtained from patients with chronic autoimmune diseases and healthy people. The multidimensionality of the questionnaire indicates the need for a summary of several components into subscales.

## Abbreviations

α: Alpha; CD: Crohn’s disease; CDAI: Clinical disease activity index; Hba1c: Glycosylated haemoglobin; HBI: Harvey Bradshaw Index; OB-Quest: Occupational balance-questionnaire; PSI: Person separation index; PCA: Principal component analysis; DIF: Differential item functioning; RA: Rheumatoid arthritis; SF-36: Short-Form 36-items Health Survey; SLE: Systemic lupus erythematous; SSc: Systemic sclerosis; T1D: Diabetes mellitus type one; SLEDAI: Systemic lupus erythematosus disease activity index; RODNAN: Rodnan skin score.

## Competing interests

There are no declared competing interest of the authors.

## Authors’ contributions

MD and TS were involved into conception and design, the acquisition of data, the analysis and interpretation of data, wrote the draft manuscript, and gave final approval of the manuscript. GS contributed to the conception and design of the study, assisted the data acquisition, analysis and interpretation, and the draft version, and finally gave advice on editing of manuscript. AK-W, VF-M, CD, and JS, gave substantial contributions to conception and design, supported the acquisition of the data, have been involved in revising the draft manuscript critically, and finally approved the manuscript considered for publication. BP was involved into conception, design of the study, and item development, supported the analysis and interpretation of the data, contributed substantially to the draft manuscript and approved the final version. AB was involved into the conception and design, item development, the acquisition and the interpretation of the data, the writing of the draft manuscript and gave final approval of the manuscript. MAS was involved into the acquisition and the interpretation of the data, the writing of the draft manuscript and gave final approval of the manuscript. The authors have taken an active part in the study and take responsibility for its contents. The FWF had no influence on the manuscript. All authors read and approved the final manuscript.
